# Use of Diagnostic Laparoscopy for Identification of Bilateral Noncommunicating Hydroceles in an Infant with Right-Sided Abdominoscrotal Mass and Left-Sided Scrotal Mass

**DOI:** 10.1155/2017/8602584

**Published:** 2017-07-31

**Authors:** Kian Asanad, Pooya Banapour, Monica Metzdorf

**Affiliations:** ^1^David Geffen School of Medicine, University of California, Los Angeles, 10833 Le Conte Ave, Los Angeles, CA 90095, USA; ^2^Department of Urology, Kaiser Permanente Los Angeles Medical Center, 4900 Sunset Blvd, Los Angeles, CA 90027, USA

## Abstract

Infantile abdominoscrotal hydrocele (ASH) is a rare condition characterized by a dumbbell-shaped cystic mass extending from the scrotum to the abdomen. We present the case of a 4-month-old infant who presented with progressively enlarging bilateral scrotal swelling and a tense, ballotable right-sided abdominal mass with extension into the scrotum. Scrotal ultrasound revealed bilateral hydroceles but exam and ultrasound could not rule out communication. At the time of planned hydrocelectomy, initial diagnostic laparoscopy was used to identify a massive right-sided ASH extending from the internal ring to the umbilicus and a large noncommunicating left-sided hydrocele that was visible with application of pressure to the left side of the scrotum. Following confirmation of anatomy with diagnostic laparoscopy, a scrotal approach to hydrocelectomy was performed as well as bilateral orchidopexy.

## 1. Introduction

Infantile abdominoscrotal hydrocele (ASH) is a rare variant of the common pediatric hydrocele with an estimated prevalence between 0.4 and 3.1%, of which bilateral cases are exceedingly rare [1]. ASH is characterized by a large, dumbbell-shaped cystic lesion that extends from the scrotum, through the inguinal canal and internal inguinal ring, into the abdominal cavity. Clinical diagnosis is suggestive with classic palpation of the abdominal mass causing an enlarging scrotum and is often confirmed with ultrasonography. It can sometimes be difficult with large, tense hydroceles to determine if they are communicating or not. Although controversy exists regarding the optimal surgical management, a recent systematic review demonstrated an inguinal surgical approach to ASH as the most common; however a scrotal approach was associated with decreased morbidity [2]. Determination of communication in advance can help guide surgical approach. Here, we report our approach to an infant with an enlarging left-sided scrotal mass and enlarging right-sided abdominoscrotal mass.

## 2. Case Presentation

**Figure 1:**
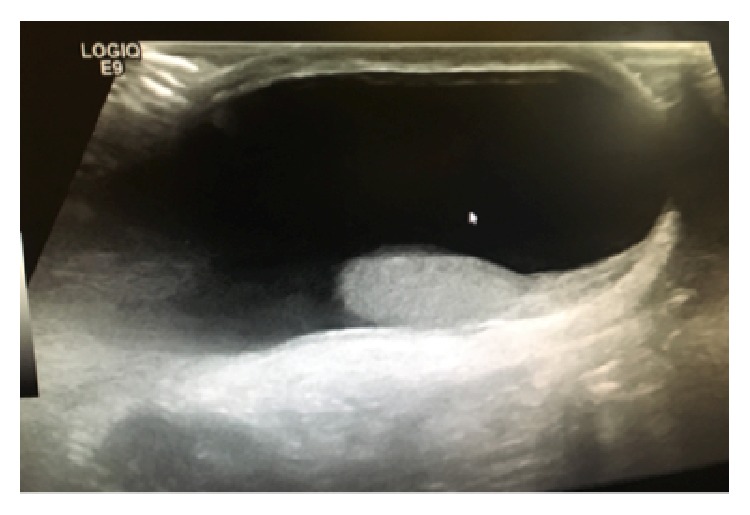
Ultrasound examination. Sagittal image of the left scrotal mass demonstrating the difficulty to appreciate the full extent of the abnormality.

The patient was a 4-month-old, full-term, otherwise healthy male infant born by spontaneous vaginal delivery who presented for evaluation of bilateral, painless, and nonerythematous scrotal swelling. The swelling was present at birth and had been progressively enlarging. There was no history of trauma. Examination revealed a tense left-sided transilluminating scrotal mass consistent with hydrocele; however it was difficult to determine if it was communicating or not. In addition, there was a much larger, palpably tense right-sided hydrocele extending into the groin and abdomen suspicious for ASH. There was obvious right scrotal enlargement with palpation of the abdomen. Testes were unable to be palpated bilaterally due to the tense nature of the hydroceles. Ultrasonography confirmed bilateral hydroceles, measuring 21.23 and 12.19 ml in the right and left scrotal sacs, respectively. However, extension of the left scrotal mass with the abdomen was not easily identified on ultrasound examination, as illustrated in Figure 1.

**Figure 2:**
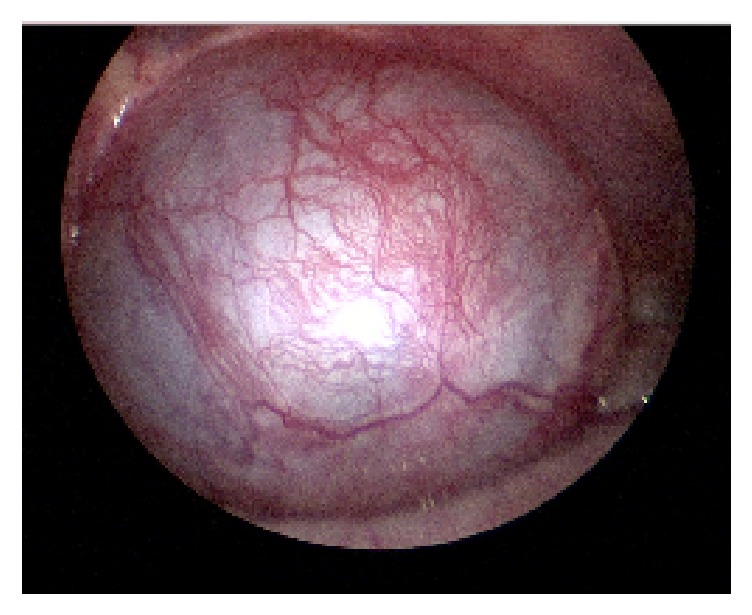
Diagnostic laparoscopy. Bulging cystic mass demonstrating a right abdominoscrotal hydrocele.

**Figure 3:**
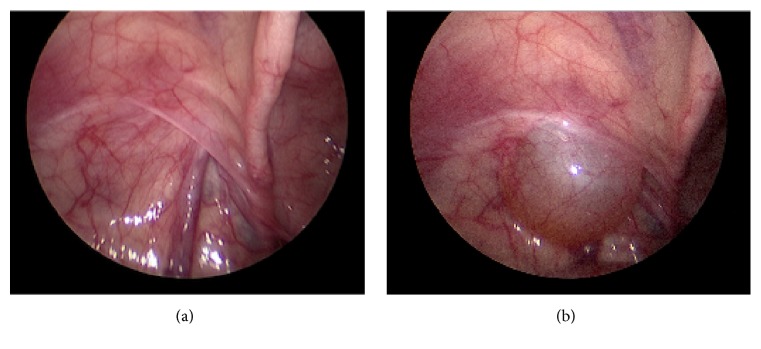
(a) Diagnostic laparoscopy. Left internal inguinal ring without palpation of the ipsilateral hemiscrotum. (b) Diagnostic laparoscopy. Left internal inguinal ring with palpation of the ipsilateral hemiscrotum.

Due to suspicion for bilateral ASH, an initial diagnostic laparoscopy was performed at the time of planned surgical repair to guide an inguinal or scrotal approach for each hydrocele. Diagnostic laparoscopy revealed a massive right-sided cystic structure extending from the internal inguinal ring displacing the colon and extending up to the level of the umbilicus, consistent with ASH (Figure 2). The left internal ring was closed and did not reveal an abdominal cystic mass (Figure 3(a)); however, with palpation of the scrotum on the left side, the hydrocele sac was seen to enlarge into the abdominal cavity (Figure 3(b)). A scrotal approach to hydrocelectomy was then performed bilaterally with removal of 150 mL and 25 mL of straw-colored fluid from right and left sacs, respectively. Bilateral orchiopexy was also performed using two-point fixation. There were no complications and at 4-month follow-up, he is doing well with no evidence of recurrence.

## 3. Discussion

Diagnosis of ASH is made predominately by physical examination with pressure applied over the abdomen causing increasing swelling in the scrotum, and vice versa. This is known as the “spring back ball sign” [2]. Ultrasonography is typically used as confirmation and evaluation of the extent of abdominal and scrotal components. In this case, there was high suspicion for right ASH but determination of communication on the left was unclear. The attending surgeon (MM) has had previous experience with ASH and has found the inguinal approach to be associated with higher recurrence and greater morbidity; therefore, confirmation of noncommunication was essential to help guide the surgical approach in this case. Although the use of CT or MRI has been previously reported in the literature to diagnose ASH [3], these diagnostic modalities were not pursued in this case. Radiation exposure from CT and the need for sedation with MRI in our facility limit our use of these modalities. In addition, while these studies can be diagnostic, they are not therapeutic and can be expensive. On the other hand, initial diagnostic laparoscopy at the time of planned repair allows for direct visualization of the abdomen with palpation of the hemiscrotum, adds only a minimal amount of additional operating room time, and helps guide an appropriate therapeutic approach. This dynamic ability is advantageous to CT or MRI, which are static imaging modalities. We use it in much the same way as diagnostic laparoscopy is used in the evaluation of nonpalpable testes.

To our knowledge, this is the first reported case where an atypical presentation suspicious for bilateral ASH was evaluated with initial diagnostic laparoscopy, rather than other imaging modalities such as CT or MRI. Although diagnostic laparoscopy carries additional surgical risks and may initially seem an invasive strategy to diagnose ASH, we find it helpful to guide our surgical approach in cases with equivocal physical exam and ultrasound findings. We agree with others who have noted increased morbidity with inguinal repair for noncommunicating hydroceles [4] and prefer scrotal hydrocelectomy in the case of ASH.

## 4. Conclusions

We report a unique case of an infant with a large, tense left hydrocele and massive right hydrocele suspicious for bilateral ASH. We believe the use of initial diagnostic laparoscopy at time of planned hydrocelectomy can be helpful to confirm the anatomy to guide the surgical approach, much in the same way as diagnostic laparoscopy is used in the evaluation of nonpalpable testes.
